# Prognostic Gene Expression Signature in Patients With Distinct Glioma Grades

**DOI:** 10.3389/fimmu.2021.685213

**Published:** 2021-09-01

**Authors:** Saadia Ait Ssi, Dounia Chraa, Khadija El Azhary, Souha Sahraoui, Daniel Olive, Abdallah Badou

**Affiliations:** ^1^Cellular and Molecular Pathology Laboratory, Faculty of Medicine and Pharmacy of Casablanca, Hassan II University, Casablanca, Morocco; ^2^Team Immunity and Cancer, Centre de Recherche en Cancérologie de Marseille (CRCM), Inserm, 41068, CNRS, UMR7258, Institut Paoli-Calmettes, Aix-Marseille University, UM 105, Marseille, France; ^3^Mohammed VI Center of Oncology, CHU Ibn Rochd, Faculty of Medicine and Pharmacy of Casablanca, Hassan II University, Casablanca, Morocco

**Keywords:** glioma, tumour-infiltrating immune cell, biomarker, immune response, prognosis

## Abstract

**Background:**

Glioma is the most common type of primary brain tumor in adults. Patients with the most malignant form have an overall survival time of <16 months. Although considerable progress has been made in defining the adapted therapeutic strategies, measures to counteract tumor escape have not kept pace, due to the developed resistance of malignant glioma. In fact, identifying the nature and role of distinct tumor-infiltrating immune cells in glioma patients would decipher potential mechanisms behind therapy failure.

**Methods:**

We integrated into our study glioma transcriptomic datasets from the Cancer Genome Atlas (TCGA) cohort (154 GBM and 516 LGG patients). LM22 immune signature was built using CIBERSORT. Hierarchical clustering and UMAP dimensional reduction algorithms were applied to identify clusters among glioma patients either in an unsupervised or supervised way. Furthermore, differential gene expression (DGE) has been performed to unravel the top expressed genes among the identified clusters. Besides, we used the least absolute shrinkage and selection operator (LASSO) and Cox regression algorithm to set up the most valuable prognostic factor.

**Results:**

Our study revealed, following gene enrichment analysis, the presence of two distinct groups of patients. The first group, defined as cluster 1, was characterized by the presence of immune cells known to exert efficient antitumoral immune response and was associated with better patient survival, whereas the second group, cluster 2, which exhibited a poor survival, was enriched with cells and molecules, known to set an immunosuppressive pro-tumoral microenvironment. Interestingly, we revealed that gene expression signatures were also consistent with each immune cluster function. A strong presence of activated NK cells was revealed in cluster 1. In contrast, potent immunosuppressive components such as regulatory T cells, neutrophils, and M0/M1/M2 macrophages were detected in cluster 2, where, in addition, inhibitory immune checkpoints, such as PD-1, CTLA-4, and TIM-3, were also significantly upregulated. Finally, Cox regression analysis further corroborated that tumor-infiltrating cells from cluster 2 exerted a significant impact on patient prognosis.

**Conclusion:**

Our work brings to light the tight implication of immune components on glioma patient prognosis. This would contribute to potentially developing better immune-based therapeutic approaches.

## Introduction

Glioma is the most common type of primary brain tumors in adults, with the most aggressive form known as glioblastoma. Glioma patients are known to be extremely resistant to chemotherapy. Indeed, such a trait is the leading cause of cancer-related mortality and morbidity (1, 2). Patients with glioma have a median overall survival of <16 months (3). The World Health Organization (WHO) classified gliomas into two groups, low-grade glioma (LGG) assembling grades I and II, and high-grade glioma (HGG) of grades III and IV (4, 5). Furthermore, based on histological and molecular features, glioma patients can be stratified into oligodendroglioma or astrocytoma (LGG) and glioblastoma (HGG) with clinically relevant molecular subtypes including mesenchymal, proneural, neural, and classical. Nevertheless, these features can be associated with tumor niche (stromal cells, reactive astrocytes, tumor cells, and immune cells), patient survival, and prognosis (6, 7).

Previous studies on glioma have provided an overview regarding tumor initiation and development (8, 9). As a matter of fact, a variety of chemoattractant and inflammatory cytokines secreted by either cancer or immune cells play a pivotal role in glioma tumor progression by attracting Treg cells into the tumor microenvironment, thus inhibiting NK cell activity and hampering the activation of effector T cells. Moreover, it leads to a plasticity phenomenon that leads to the conversion of tumor-associated macrophages (TAMs) from M1 state to an anti-inflammatory M2 phenotype and T cell transition from Th1 toward a Th2 phenotype which are often associated with poor outcome (6, 10–12). Hence, the cross talk between tumor and the immune cells results in the establishment of an immunosuppressive tumor microenvironment, which promotes tumor escape and cancer progression.

Over the past few years, several immunotherapeutic approaches have been developed (13); however, clinical trials testing vaccines, cell adoptive transfer, and immune checkpoint inhibitors (ICIs) point to a lack of efficacy (14–16).

This failure has shifted the focus toward a more thorough understanding of the distinct components of the glioma microenvironment (17–19). Glioblastomas are very low-immunogenicity tumors (16), enriched with immune-suppressive T cells mainly embedded in the central nervous system (20). As a matter of fact, future treatment strategies should be designed based on an improved understanding of key immune cell interactions with glioma cells.

Recently, computational analyses of RNA-seq data have demonstrated that a glioblastoma microenvironment lacking central memory CD4 T cells or natural killer (NK) cells is further correlated with better prognosis, and the expression levels of *ICOS* and *TNFSF14* were negatively associated with clinical outcome (21). It has also been reported that the glioblastoma microenvironment contains hub genes (CCT3, OLIG2, PSMB9, TRIM21) that were associated with distinct immune cell infiltration characterized by the expression of immune checkpoints and are further involved in cancer development and progression in patients with high-grade glioblastoma (22–24). In low-grade glioma, the expression level of transforming growth factor beta 1 (*TGF-β*) and programmed cell death ligand 1 (*PDL1*) was positively correlated with immune risk score, and prognostic hub genes that were positively correlated with immune cell infiltration have also been revealed (25, 26). Notably, T follicular helper (TFH) cells, activated NK cells, and macrophages have been demonstrated to be independent predictors for malignant transformation in low-grade glioma (27). Furthermore, it has been suggested that the expressions of JAK3, IL2Rß, and CD3ε are further associated with the presence of B cell memory and CD8 T cells, which adversely impact immune cell response in the tumor microenvironment (28). As a matter of fact, Qiu et al. recently demonstrated, through their PROMISE model, differences in immune cell abundance between risk groups (29). Although innate immune cells such CD8 T cells were upregulated and further associated with good prognosis, a downregulation of TFH cells was observed in the high-risk group of glioma patients (29). Zhong et al. demonstrated that resting NK cells, CD8+ T cells, TFH cells, gamma delta T cells, and M0 macrophages were negatively related to prognosis. However, their proportion was significantly related to patient’s age and sex (30).

Nevertheless, these studies assessed low-grade glioma or glioblastoma patients individually and did not consider both subtypes as a whole to identify striking resemblance and active involvement of immune genes in glioma patients.

Here we assessed the presence of two distinct groups of glioma patients. The first group, defined as cluster 1, was associated with better patient survival, whereas the second group, cluster 2, exhibited a poor survival. Interestingly, a strong presence of activated NK cells was revealed in cluster 1. In contrast, potent immunosuppressive components such as regulatory T cells, neutrophils, and M0/M1/M2 macrophages were detected in cluster 2, where inhibitory immune checkpoints, such as cytotoxic T-lymphocyte-associated protein 4 (*CTLA4*), lymphocyte-activation gene 3 (*LAG3*), and T cell immunoglobulin mucin 3 (*TIM3*), were also significantly upregulated. Finally, Cox regression analysis further corroborated that tumor-infiltrating cells from cluster 2 exerted a significant impact on patient prognosis. Our results pinpoint that the immune response type impacts the clinical outcome of glioma patients.

## Materials and Methods

### Data Analysis From TCGA and Independent Datasets

**Figure 1:**
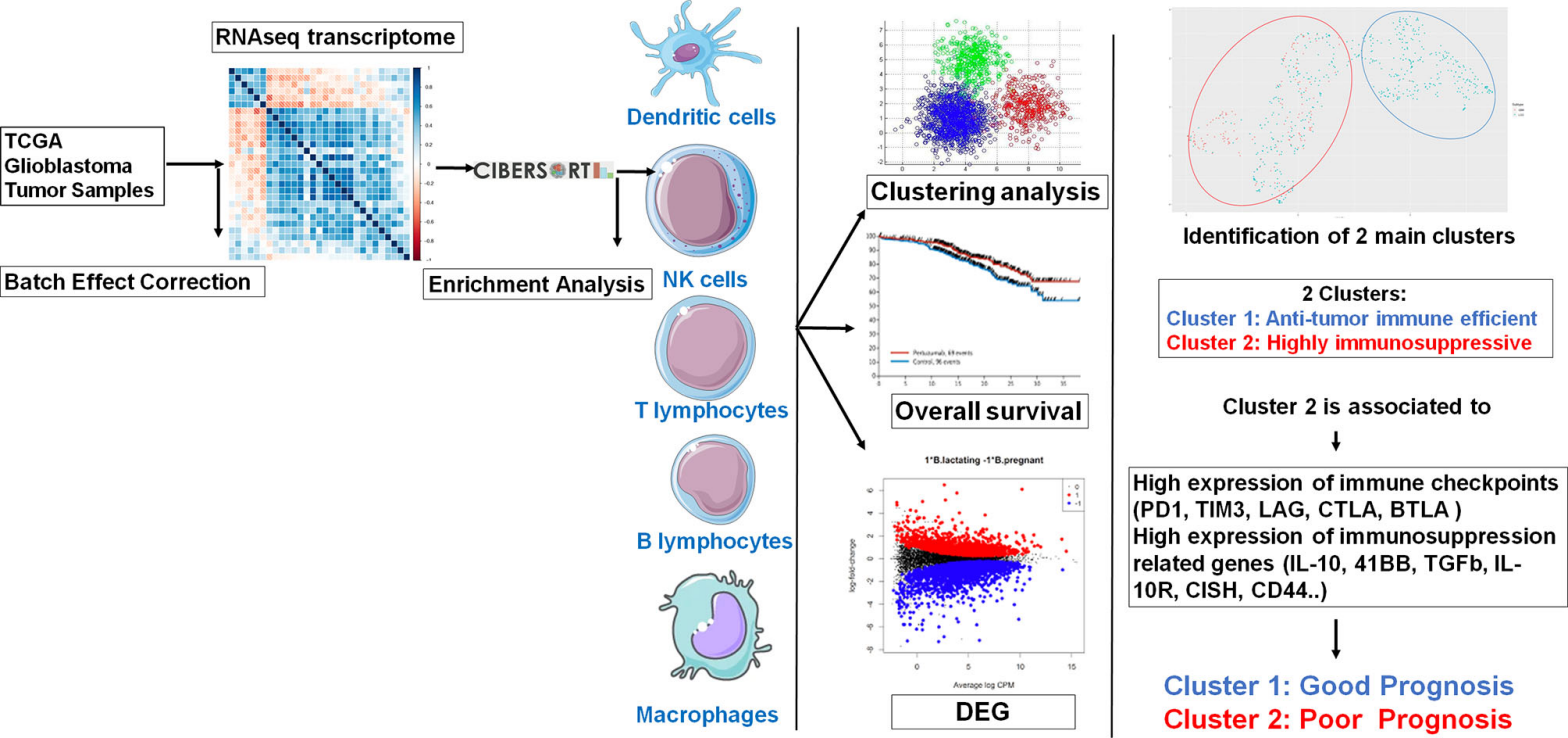
Graphical abstract.

The workflow of our study is shown in **Figure 1**. Molecular data, including mRNA expression of LGG (n = 516) and GBM (n = 154) patients, were downloaded from The Cancer Genome Atlas (TCGA) data portal (https://www.cbioportal.org/). Clinical data related to glioma patients were downloaded altogether with matrix mRNA gene expression.

### Quantification of Immune Signature Enrichment Levels in Glioma Patients

We analyzed 22 immune signatures (e.g., CD8+ T cells, CD4 naive T cells, Treg cells, naïve B cells, memory B cells, plasma cells, CD4 memory resting T cells, CD4 memory activated T cells, follicular helper T cells, gamma delta T cells, resting NK cells, activated NK cells, monocytes, macrophages M0/M1/M2, resting dendritic cells, activated dendritic cells, resting mast cells, activated mast cells, eosinophils, neutrophils). The enrichment level of this immune signature in glioma samples was quantified as the mean expression level of immune signature marker genes. We, therefore, used CIBERSORT (https://cibersort.stanford.edu/index.php) which estimates the relative fraction of each cell type in the signature matrix, such that the sum of all fractions is equal to 1 for a given mixture. The absolute immune fraction score was estimated by the median expression level of all genes in the signature matrix divided by the median expression level of all genes in the mixture.

### Hierarchical Clustering

To compare distinct immune cell infiltration genes in glioma patients, unsupervised hierarchical clustering was performed to group the 658 patients into two subgroups termed cluster 1 (n = 293) and cluster 2 (n = 365).

### Global Gene Expression Analysis

Differential gene expression (DGE) analysis with RNA-seq data was performed using the R package.

An average raw read count for each gene >1 was applied to determine candidate genes that were reasonably expressed. The expression fold change (FC) denotes upregulation or downregulation according to the FC value. Subsequently, log FC, log CPM, p-value, and the corresponding false discovery rate (FDR) were all reported by the R package. FDR < 0.05, log CPM > 1, and |log FC| > 2 were set as inclusion criteria for DEG selection.

### Association of Immune Signature Enrichment Levels With Various Molecular Features

R Programming Environment (version 3.2.5) and Bioconductor were used for preprocessing and data analysis. For data filtering and quality control, the following methods were used. The median rank scores were identified in the dataset, and quartile normalization was performed. Data were visualized as Uniform Manifold Approximation and Projection (UMAP) which engage different parameters such as the distance between points and number of neighbors for a better reduced dimensional representation. We identified the upregulated genes that were significantly associated with immune signature enrichment levels (ISELs) using the Mann–Whitney U test.

### Gene-Set Enrichment Analysis

We used FunRich to identify the KEGG pathways that were significantly associated with the genes having an important expression correlation with the immune signature using a threshold of FDR < 0.05.

### Survival Analysis

We compared the overall survival (OS) between clusters and in the glioma cohort. We used Kaplan–Meier survival curves to show the survival time differences and the log-rank test to assess the significance of survival time differences. Statistical analyses were carried out with GraphPad Prism 5.0 software (GraphPad Software, Inc., La Jolla, CA, USA). Single comparisons between two groups were performed with Student’s t-test.

Univariate and multivariate Cox regression analyses were used on tumor-infiltrating immune gene signature and clinical parameters, including IDH status and age in TCGA cohorts.

Cox regression analyses were used to evaluate the prognostic effect of the immune signature on glioma patient prognosis and were performed using the R Programming Environment (version 3.2.5) and Bioconductor. To evaluate the difference between groups, the hazard ratios HR ≥ 1 and p-value of ≤0.05 were considered significant.

## Results

### Independent Immunostimulatory and Immunosuppressive Profiles of Glioma Patients

**Figure 2:**
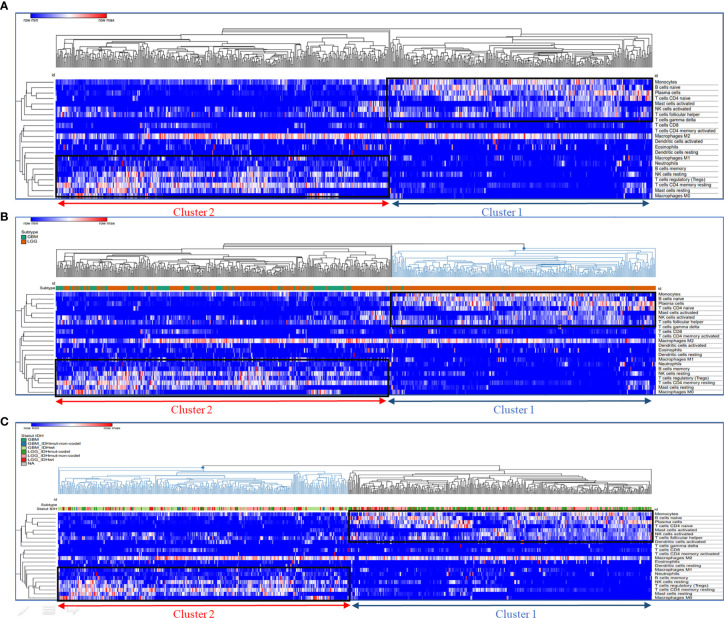
Segregation of enriched immune cell subsets by unsupervised hierarchical clustering. Hierarchical clustering of immune cell frequency in glioblastoma samples. RNA sequencing (RNA-Seq) gene expression data was analyzed with CIBERSORT to quantify the amount of different immune cells in the TCGA glioblastoma samples. Hierarchical clustering of these quantities after normalization is shown in the heat map. **(A)** Hierarchical clustering of immune cell frequency in glioblastoma samples, **(B)** Glioma Subtype groups (GBM and LGG) localisation in cluster 1 and 2; **(C)** Glioma IDH mutation status localisation in cluster 1 and 2. The cluster of samples enriched in immunosuppressive immune cells are represented in cluster 2 (red); the cluster of samples enriched in activated immune cells are represented in cluster 1 (blue). Hierarchical clustering was performed using Euclidean distance and complete linkage methods.

To identify the role of distinct tumor-infiltrating immune cells in glioma patients, a transcriptomic dataset from the Cancer Genome Atlas (TCGA) cohort (153 GBM and 512 LGG patients) was analyzed in this study. A pretreatment step of glioma transcriptomic data was carried out by emerging LGG and HGG databases, followed by a harmonization step. The unsupervised hierarchical clustering after the enrichment analysis enabled us to demonstrate the presence of two distinct clusters (groups of patients). The first group defined as cluster 1 was characterized by an immunostimulatory profile gathering monocytes, naïve B cells, plasma cells, naïve CD4+ T cells, activated mast cells, NK cells, and T follicular helper cells, whereas the second group (cluster 2) was enriched with immunosuppressive immune cells such as neutrophils, M0/M1/M2 macrophages, regulatory T cells (Tregs), resting mast cells, resting NK cells, resting CD4 memory T cells, and memory B cells (**Figure 2A**).

We further integrated clinicopathological parameters such as IDH status and glioma subtypes (LGG and GBM) to this hierarchical clustering, to assess whether the two clusters were correlated with these parameters. As shown in the hit map (**Figures 2B, C**), cluster 1 contains mainly LGG patients with only six GBM patients inside. However, the majority of GBM patients were grouped in (cluster 2). Moreover, a greater part of LGG patients with IDH mutant and 1p19q codeletion (codel) were gathering in cluster 1. Further, LGG patients with IDH wild-type or IDH mutant with 1p19q non-codeletion (non-codel) were likewise distributed within two clusters.

In order to confirm these results, we used high-dimension reduction algorithm that is based on manifold learning and nonlinear dimensionality reduction, used here as an effective preprocessing step to boost the performance of clustering. These results were confirmed either by increasing the distance between points (near neighbors) or by adding UMAP parameters (Canberra, Cosine, Dice) that control the balance of local *versus* global structure in our data. Indeed, we could observe the same clusters, which clearly suggest that this clustering depends on patients’ immune profiles rather than clinicopathological parameters (**Figures S1A, B** and **Figures S2A, B** **)**.

### Difference in Immune Cell Abundance Within the Clustered Patients

**Figure 3:**
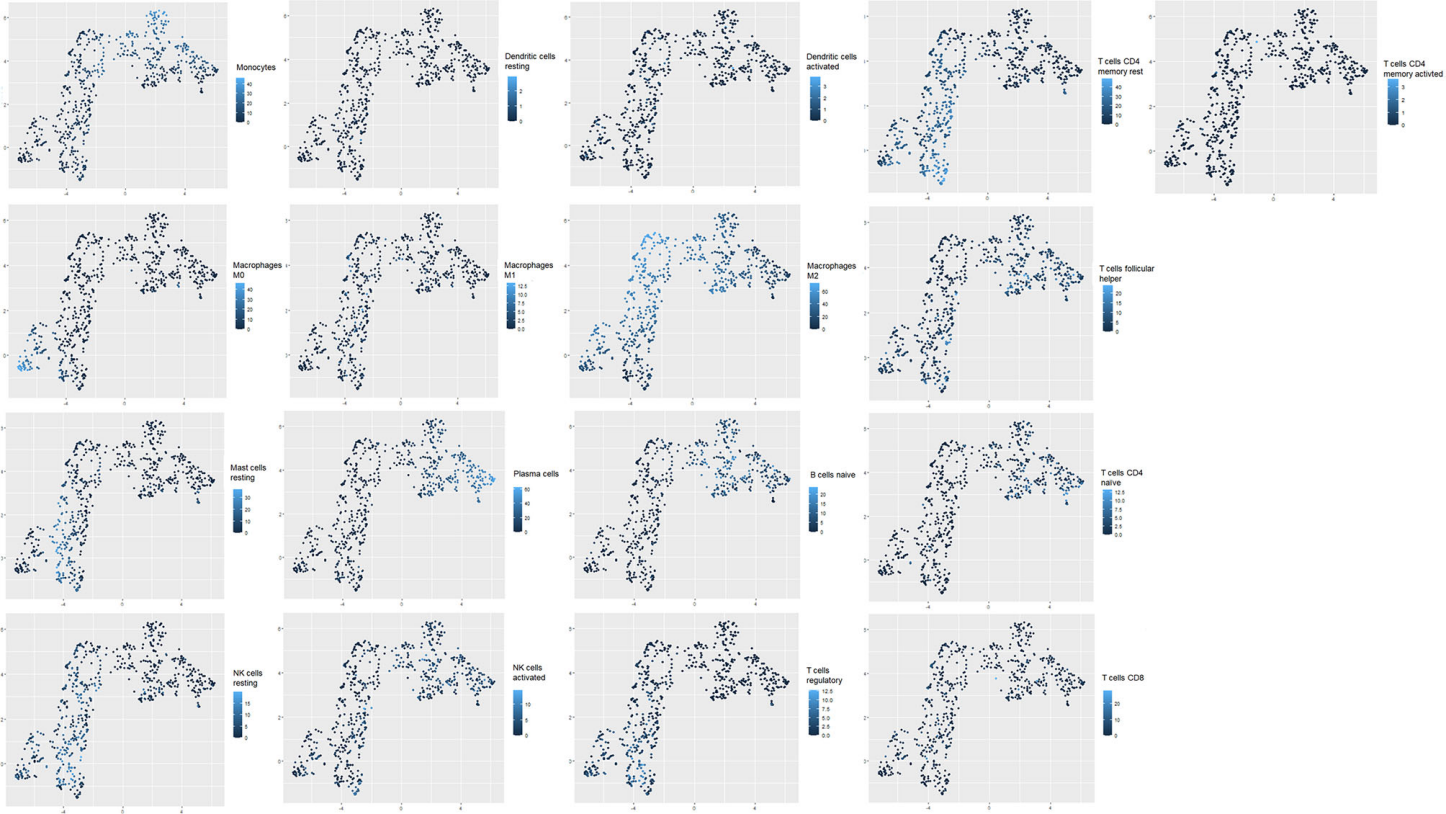
Uniform Manifold Approximation and Projection (UMAP) plots of transcriptomic profiles from glioblastoma datasets. Clustering of tumor-infiltrating immune cell populations (T regulatory cells, dendritic cells, macrophages M0, M1, and M2, CD8 T cells, activated CD4 T cells, activated NK cells, resting NK cells, B cells, T follicular helper cells); intensity of immune cell frequency within the clustered patients (cluster 1 and cluster 2). Light blue reflects a high enrichment intensity, and dark blue is associated with low infiltration intensity.

**Figure 4:**
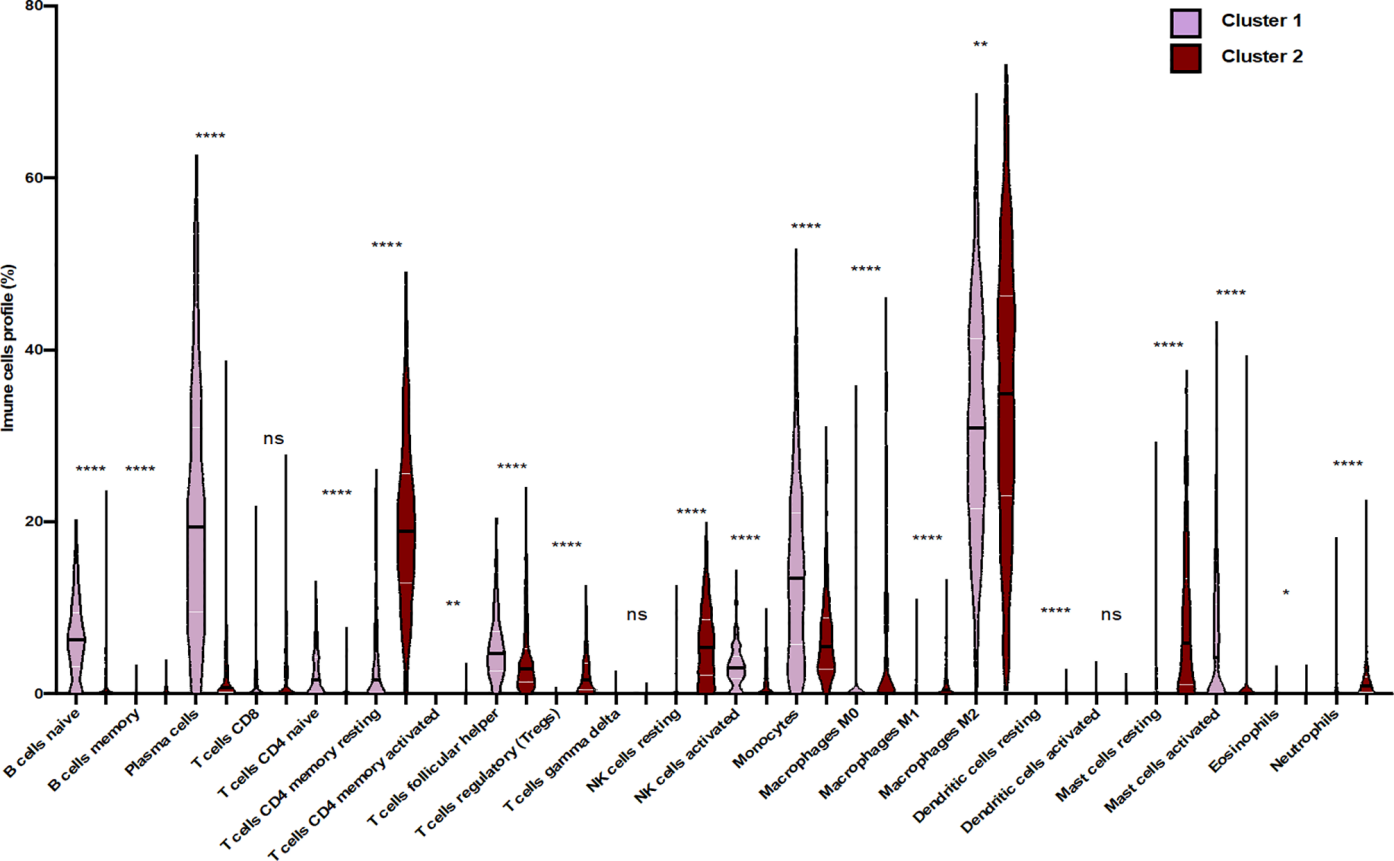
Differential distribution of immune cell proportion within the two clusters of glioma patients. Violin plots present the distribution of each immune subset. Violin plots show median values (black horizontal lines) in glioblastoma data. The Wilcoxon test shows statistically significant differences in the immune cell subtypes between the two clusters (*p < 0.05, **p < 0.01, ****p < 0.0001, ns, statistically no significant).

To confirm that unsupervised clustering could mostly be driven by the immune enrichment profile in patients with gliomas associated with the tumor microenvironment, we used this time UMAP to score the intensity of each infiltrated immune cell population within the clustered patients (**Figure 3**). As a matter of fact, we observed more naive B cells, plasma cells, naive CD4 T cells, TFH cells, monocytes, activated mast cells, and activated NK cells in cluster 1 than in cluster 2. When compared to cluster 1, cluster 2 seems to gather more less memory B cells, CD4 memory resting T cells, CD4 memory activated T cells, regulatory T cells, resting NK cells, M0/M1/M2 macrophages, resting dendritic cells, resting mast cells, and neutrophils. According to the Wilcoxon test, there were statistically significant differences in the immune cell subtypes between the two clusters (p < 0.05; FC > 2) (**Figure 4**), which confirm the immunosuppressive trait of cluster 2 relative to cluster 1. Indeed, these results highlight the weight of the immune profile on patients.

### Association of Immune Checkpoints Overexpression With Immunosuppressive Status

Considering the critical role of the immune checkpoint inhibitors such as programmed-death receptor-1 (*PD-1*) and cytotoxic T lymphocyte antigen-4 (*CTLA-4*), not only in the negative regulation of T-cell activity but also in modulating T-cell migration into tissues (31), we aimed to compare the expression level of immune checkpoints between cluster 1 and cluster 2. As illustrated in **Figure 4**, *PDL-1*, *CTLA-4*, *TIM-3*, *LAG-3*, band T lymphocyte-associated (*BTLA*), inducible T cell costimulator (*ICOS*), inducible T cell costimulator ligand (*ICOSL*), and GATA-binding protein 3 (*GATA-3*) expressions were significantly higher in cluster 2 than in cluster 1 (p value <0.05; FC > 2).

**Figure 5:**
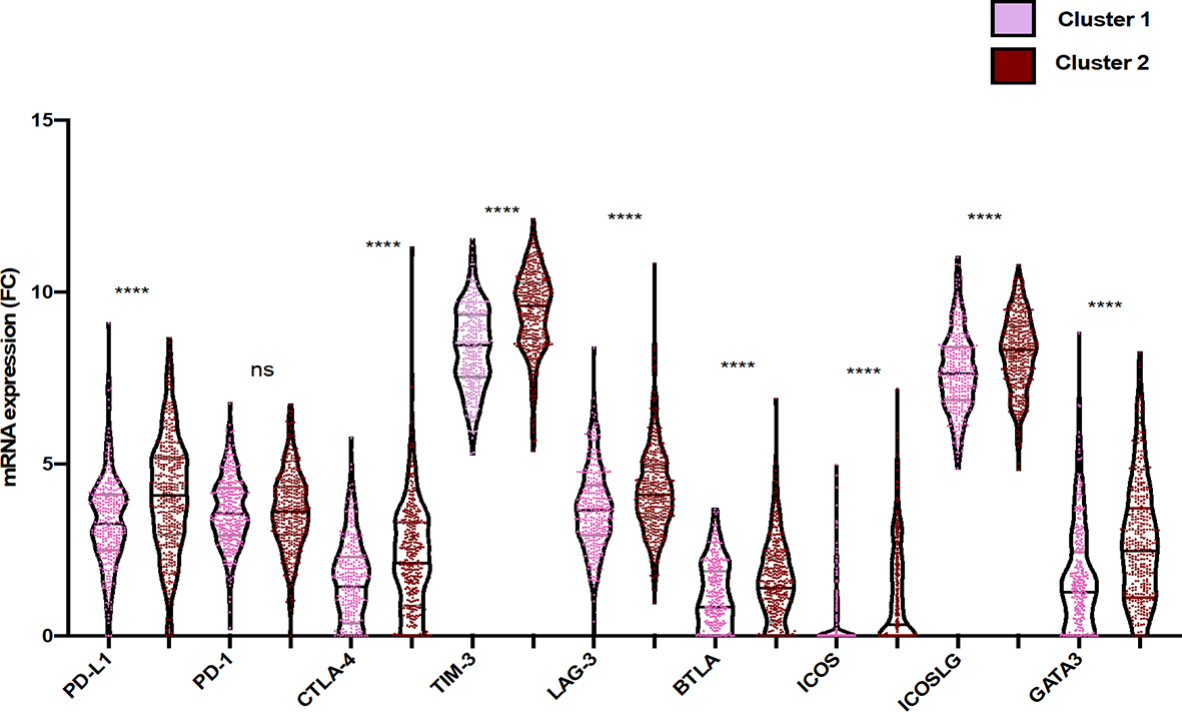
Differential gene expression of immune checkpoint within the two clusters of glioma patients. Violin plots represent the relative mRNA gene expression in the two clusters (pink: cluster 1; red: cluster 2). Each dot represents a sample. Violin plots show median values (black horizontal lines) in glioblastoma data. The two-tailed independent test was applied. p values of differences between groups are shown above (p < 0.05, ****p < 0.0001, ns, statistically no significant).

Furthermore, a significant correlation between the expressions of *PD-1*, *BTLA*, and *ICOS* has been observed. In addition, our results showed that infiltration of immune cells such as CD8 T cells was associated with the expression of *GATA3*. Activated CD4 memory T cells and macrophages (M1/M2) were importantly correlated with expressions of *BTLA* and *ICOS [r* (*-1 to +1*), *p<0.05]*. Moreover, regulatory T cells were highly correlated with CD4 memory T cells, resting NK cells, macrophages (M0), and resting mast cells. Likewise, activated NK cells were associated with plasma cells, B cells naïve, follicular helper T cells, eosinophils, and activated mast cells (**Figure 5** and **Figure S3**).

Our results suggest that NK cells, plasma cells, naïve B cells, follicular helper T cells, eosinophils, and activated mast cells cooperatively exhibit a balance of inhibitory and activating signals in cluster 1 where the cells expressed fewer immune checkpoints.

### Relevant Activator and Inhibitor Markers to Guide Characterization of Glioma Clusters

**Figure 6:**
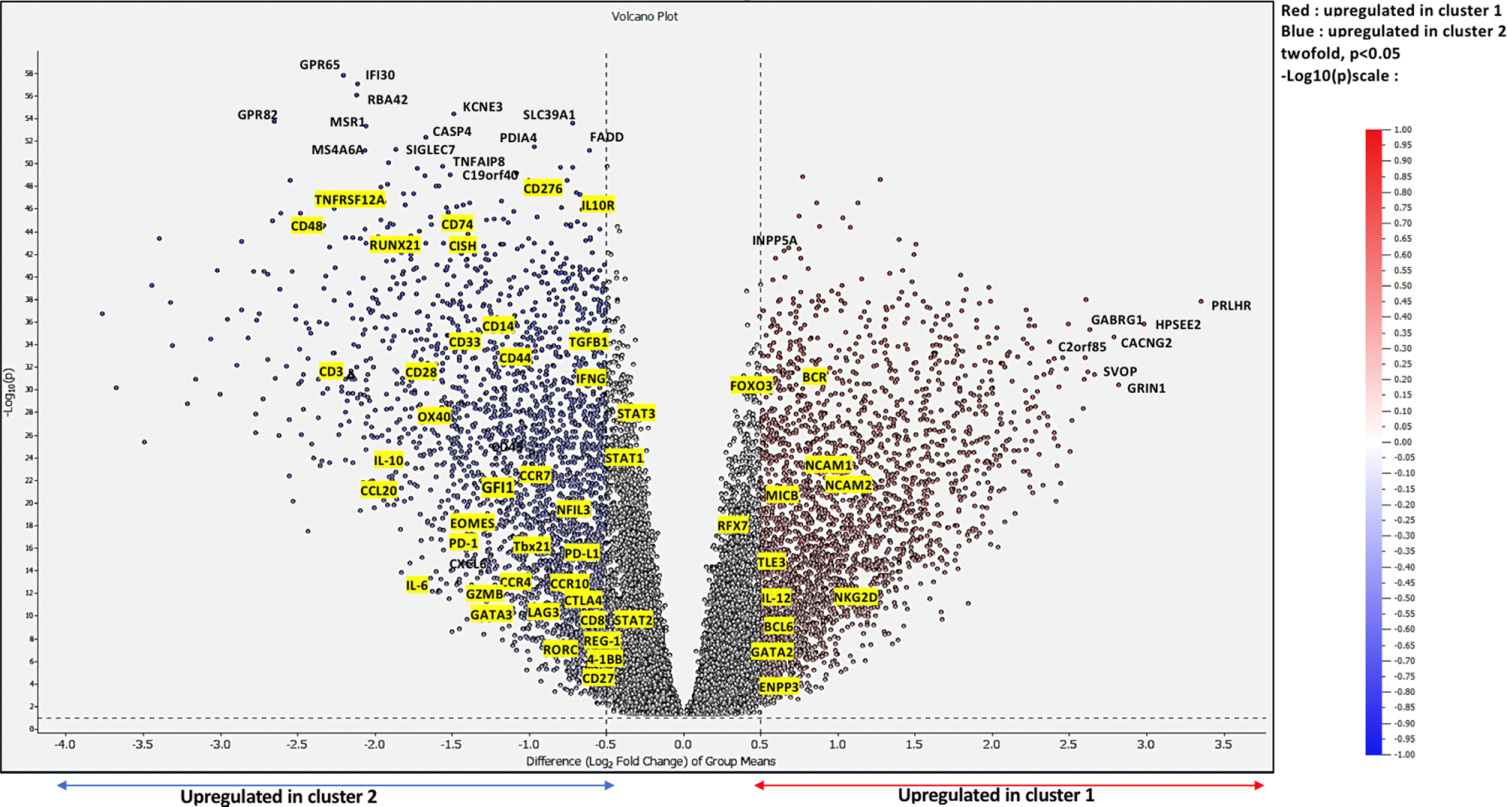
Differentially expressed genes at a Bonferroni-corrected p-value. Volcano plot showing upregulated genes in cluster 1 shown in red *vs.* upregulated genes in cluster 2 shown in blue while non-DE genes are in black.

To further deepen our investigation on the identified clusters, we performed differential expression gene (DEG) analysis based on 10,000-gene fold change expression within each cluster. As a matter of fact, we used as a fold change cutoff FC > 0.5 and FC < -0.5; all downregulated genes and upregulated genes are shown in the volcano plot (**Figure 6**).

Our results demonstrated that, on the one hand, genes such as neural cell adhesion molecule 1 (NCAM1), neural cell adhesion molecule 2 (NCAM2), MHC class I polypeptide-related sequence B (MICB), killer cell lectin-like receptor K1 (*NKG2D*), and interleukin 12 (IL-12) described as markers of activation were exclusively upregulated in cluster 1. On the other hand, the expressions of genes such as *TGFβ*, *IL-10*, interleukin 6 (*IL-6*), *GATA3*, regenerating family member 1 (*REG-1*), RAR related orphan receptor C (*RORC*), *LAG3*, *CTLA4*, cluster of differentiation 276 (*CD276*), cytokine-inducible SH2-containing protein (CISH), and cluster differentiation (*CD44*), further associated with the inhibition of T cell effector activity, were upregulated in cluster 2, where immunosuppressive populations are strongly enriched, as we previously demonstrated. Indeed, we could clearly observe the consistent link between the upregulated genes in each immune cluster component.

### Immune Cell-Dependent Prognostic Drivers of Glioma Patients’ Survival

**Figure 7:**
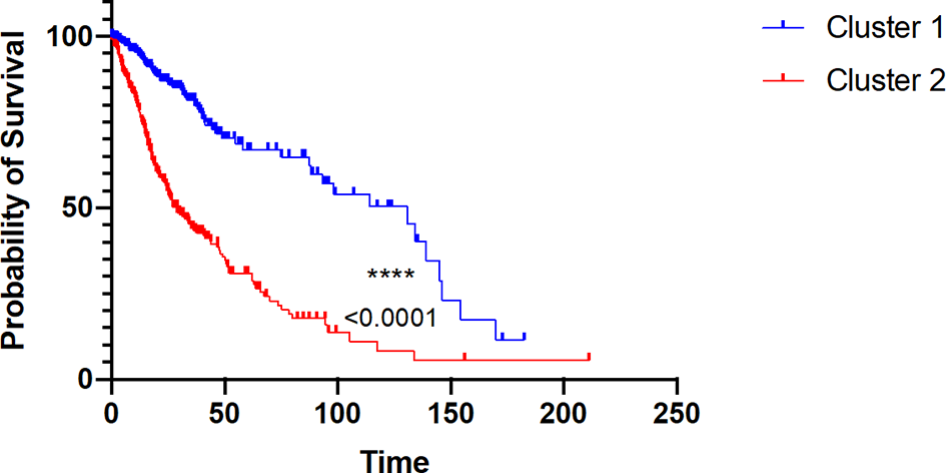
Kaplan–Meier survival probability of the two clusters of glioma patients. Red curve represents overall survival (OS) of cluster 2 patients. Blue curve represents overall survival (OS) of cluster 1 patients, with p value < 0.0001.

To assess whether the difference in immune components and other upregulated genes within cluster 1 and cluster 2 would reliably affect patient prognosis, we performed Kaplan–Meier (K-M) analysis between the two clusters. As a matter of fact, cluster 2 was strikingly associated with poor survival (at 50% of overall survival (OS), the survival median was at roughly 25 months) (p < 0.0001), which fits our results (**Figure 7**).

Then, we conducted a univariate Cox regression analysis based on clinical features and tumor-infiltrating immune cell parameters of cluster 2 patients for assessing prognostic factors that independently influence glioma patient survival (**Table 1**).

**Table 1:** Multivariate analysis of the mean tumor-infiltrating immune cells in cluster 2 and glioma clinicopathologic features (IDH status, age).

TIICs and clinicopathologic parametres	Ex (-coefficient)	Ex (coefficient)	(95% CI)	
			Lower	Upper
**IDH status**	2.535e + 00	3.945e - 01	0.33134	4.698e - 01
**Age**	1.179e + 00	8.480e - 01	0.63580	1.131^e^ + 03
**Regulatory T cells**	1.305e - 01	7.662e + 00	0.00735	7.988^e^ + 03
**Neutrophils**	1.891e - 02	5.288e + 01	0.15337	1.823^e^ + 04
**Macrophages M0**	3.537e - 02	1.974e + 04	6.53557	1.223^e^ + 02
**Macrophages M1**	5.065e - 5	1.974e + 04	16.30275	2.391^e^ + 07
**Macrophages M2**	5.666e - 01	1.765e + 00	0.66034	4.718e + 00

Concordance: 0.786 (se = 0.018) p-value<2e-6.

Likelihood ratio: 154.8 p-value<2e-6.

Wald test: 158.8 p-value<2e-6.

Score (log-rank test):179.8 p-value<2e-6. e = Exponential, i.e., e-01 = 10^-1^.

However, we have demonstrated by multivariate Cox regression analysis that regulatory T cells (Treg), neutrophils, and macrophages M0/M1/M2 are key drivers of cluster 2 patient survival, since the Ex coefficients were respectively 7.662e^+00^, 5.288e^+01^, 1.974e^+04^, 1.974e^+04^, and 1.765e^+00^; p < 2 e^-6^; and hazard ratio (HR) ≥ 1 (**Table 1**). These results might shed light on the importance of immune infiltrate when establishing patient diagnosis, a key feature that might influence glioma patient overall survival.

## Discussion

The tumor microenvironment plays an important role in glioma pathogenesis. Here, we provided the immune signature, which might impact glioma patient prognosis. Using LM22 CIBERSORT, followed by unsupervised hierarchical clustering and UMAP dimensional reduction algorithms, we identified two clusters in glioma patients. These clusters were associated with immune infiltration character which is basically related to some specific expressed genes inside the tumor microenvironment and further associated with either good or poor prognosis. Thus, even though clinico-pathological parameters were added to the unsupervised clustering, those two groups of patients were identified independently of other prognostic parameters such as IDH status and glioma subtype. Then, we employed different methods to confirm our unsupervised clustering. Indeed, our data highlight the strength of the defined immune cells shown in terms of hierarchically clustering our patients.

As a matter of fact, we found that cluster 1 was highly correlated with naive B cell, plasma cell, naive CD4 T cell, T follicular helper cell, NK cell, and monocyte infiltration. It has been previously reported that B cell infiltration in melanoma, breast, and lung cancers was positively correlated with a better survival (32, 33). Indeed, naive B cells and plasma cells can enhance an efficient immune response by activating cytotoxic T cell response (34). Furthermore, B cells have been described as a central player in the response to immune checkpoint blockade in patients with metastatic melanoma and renal cell carcinoma, through the activation of T cells and the generation of IgG subclasses of antibodies (35–37). Moreover, combining radiation, PD-L1 inhibition, and B cell-based vaccine that consists of *4-1BBL*+B cell activation promoted glioma cell regression by 80% in mice (38).

Besides, the presence of cytotoxic immune cells such as NK cells is associated with a good prognosis in glioma patients (39). Considering the role of NK cells in glioma immunosurveillance, many clinical trials focus on CAR-NK cells for the treatment of glioblastoma patients (40, 41).

Naïve CD4 T cells can proliferate and differentiate into an effector ThCTL subpopulation which contributes to an effective antitumor response through antigen recognition of peptides presented by MHC class II molecules following CTLA4 blockade (42). Yet, it has been reported that naïve T cells are missing in the glioblastoma microenvironment due to their sequestration in the bone marrow (43). Our data consolidate the thought that cluster 1 is enriched by antitumoral cell subsets with immunostimulatory characteristics.

Our results not only showed that cluster 2 was poorly enriched in activated and cytotoxic T cells but also underlined the high prevalence of M0 and M1 macrophages and neutrophils. Moreover, Treg cells and M2 macrophages were highly enriched in this group of glioma patients. Moreover, Treg accumulation in the tumor microenvironment was associated with antitumor T cell repression (44), poor prognosis (45), and resistance to vascular endothelial growth factor (*VEGF*) therapy in glioblastoma (46). The predominance of M2 macrophages in the glioma microenvironment has been described to be associated with poor survival and resistance to radiotherapy, whereas M1 macrophages have been reported to be less abundant in high-grade glioma and expression of M1 marker C–C motif chemokine ligand 3 (*CCL3*) was further associated with a good survival (47, 48). Moreover, clinical data suggest that neutrophils were associated with poor prognosis and tumor progression in glioma patients (49, 50). Altogether, these studies and our results come to strengthen the hypothesis that the immune infiltrate components should be taken into consideration when establishing patients’ diagnosis. These data show that cluster 2 is infiltrated by pro-tumoral cells with immunosuppressive properties.

According to the results of patients responding to anti-PD-1/anti-PD-L1 immunotherapy, Mellman and colleagues described two categories of microenvironment immune profile. The first is “immune inflamed tumor” that exhibits a preexisting antitumor immune response and provides a more favorable environment for T cell activation and renewal. The second profile is named “non-inflamed tumors” where cells are in a pausing state either in the parenchyma or in the tumor stroma and release more immunosuppressive cytokines (51). Our observations demonstrated that the immune components of cluster 1 patients presented similar features to “immune inflamed tumor” such as the abundance of *IL-12*, *MICB*, *NCAM1*, *NCAM2*, and *NKG2D* while the second group (cluster 2) seemed to exhibit some of the “non-inflamed tumors” characteristics such as the expressions of *TIM3*, *PDL1*, *IL-10*, and *TGF-β*.

Differential expression genes revealed the top genes that are, according to the literature, distinctively associated with an antitumor immune response in cluster 1 (*MICB*, *NKG2D*, *NCAM1*, *NCAM2*, and *IL-12*). As a matter of fact, *MICB* overexpression by tumor cells activates cytotoxic lymphocyte functions such as lysis and elimination of cancer cells through *NKG2D* receptor activation (52, 53). Furthermore, *NKG2D* receptor and *NCAM* upregulation triggers cytotoxic effector functions and provides costimulatory signals for NK cells, CD8 T cells, and ɣϨT cells (54–56). Moreover, *IL-12*, which plays a key role in changing tumor microenvironment composition from one that contains less differentiated Th0 cells to one that has more inflammatory Th1 cells, was also upregulated (57). Besides, *TLE3* upregulation may also induce cell cycle arrest and tumor growth suppression *via* inhibition of *MAPK* and *AKT* pathways (58, 59). On the other hand, *GATA2* upregulation induces cell surface *PDL-1* and *PDL-2* expression in brain tumors, and its inhibition has been reported to stimulate chemotherapy-mediated apoptosis in human AML cells overexpressing *GATA2* (60, 61). Further, *BCL6* overexpression has pro-survival and proliferative activities of glioma cells; its expression in monocytes/macrophage lineage reduces the antitumor immune response and increases the immunosuppressive microenvironment. It has been demonstrated recently that *BCL6* deletion in Treg cells significantly inhibited tumor progression (62–64).

Most interestingly, upregulated genes in cluster 2 (*TGFβ*, *IL-10*, *IL-6*, *GATA3*, *REG-1*, *RORC*, *LAG3*, *CTLA4*, *CD276*, *CISH*, and *CD44*) were related to an activated regulatory T-cell phenotype. Indeed, transcription factors such as *GATA3* and *RORC* are further related to the differentiation of CD4 T cells into Th17 that plays a pivotal role in regulatory T cell generation and recruitment (61–64). TGF-β and IL-10 are anti-inflammatory, which, produced by many cell types, play a crucial role in inhibiting the antitumor immune response in glioma (65–67) by activating the polarization of M0 into M1 or M2 macrophages (68–70). Strikingly, *CD276* and nuclear factor interleukin 3-regulated (NFIL3) overexpression is capable of inducing T cell immunosuppression (71, 72). Moreover, the co-expression of PD*-1* with other inhibitory receptors, such as *PDL-1*, *TIM-3*, *LAG3*, and *CTLA-4*, induces effector T cell exhaustion (73, 74). It has also been reported that the PD-1/PDL-1 pathway is significantly correlated with the most aggressive histological subtype of glioma (75, 76). This could reflect the importance of the complex mechanisms established by the tumor microenvironment to suppress the antitumor immune response.

Our survival results highlight the importance of immune parameters in the outcomes of glioma patients and could further be considered as the most suitable predictive feature when adopting new treatment strategies. So far, clinicopathological and genetic features could not be used as predictive parameters in glioma patients’ survival. In fact, glioma patients who have been grouped based on similar clinical characteristics could still present contradictory outcomes (25, 77).

Cox regression results indicated that macrophages M0/M1/M2, neutrophils, and Treg cells might have a detrimental impact in cluster 2 patients’ survival. Our findings shed light on the importance of immune infiltration on glioma patients’ survival. Differential expression of the immune checkpoint inhibitors between cluster 1 and cluster 2 might occur due the presence of macrophages and Treg cells. Indeed, cluster 2 exhibited a high expression of immune checkpoints and was mainly enriched with macrophages M0/M1/M2, neutrophils, and regulatory T cells. In fact, these cells could be involved in the upregulation of checkpoint expression and thus the inhibition of cytotoxic T cell activities (cluster 2). Therefore, understanding the signaling pathways of these cell subsets and their interaction with immune checkpoints would provide more information to define best-suited treatment strategies for glioma patients.

Over the past decade, it has been strongly believed that glioma patients exhibit resistance to adjuvant therapy. As a matter of fact, several resistance mechanisms limit the efficiency of classical treatments (78). Moreover, glioblastoma bears characteristics that contribute to significant therapeutic resistance by preventing adequate control of the entire tumor mass by drugs. Glioblastoma therefore facilitates escape mechanisms by mediating blood–brain barrier junction proteins dysfunction (79). Understanding the different interactions that occur inside the tumor microenvironment is crucial to conceive reliable therapeutic approaches.

Our findings shed light on the importance of using some immune markers to further stratify high- and low-risk glioma patients in order to suggest the adequate treatment for each group. As a matter of fact, Treg and NK cell infiltration rates found within cluster 2 in our study further indicate that this group would probably respond to NK activation treatment or to Treg cell targeting.

Our study comes with important findings which should be further corroborated. As a matter of fact, we have used CIBERSORT to identify critical immune infiltration subtypes. However, given that LM22 involves a limited set of genes and that we still lack evidence for a direct role of each cell subtype within glioma TME, a deepened characterization of immune infiltrates in glioblastoma might be necessary to further strengthen our findings.

## Conclusion

Our work brings to light the tight implication of immune components on glioma patient prognosis. Moreover, characterizing immune subsets in the tumor microenvironment could also provide predictive factors that would contribute to potentially developing better immune-based therapeutic approaches.

## Data Availability Statement

The raw data supporting the conclusions of this article will be made available by the authors, without undue reservation.

## Author Contributions

SA analyzed the data and wrote the manuscript. DC analyzed the data and wrote and revised the manuscript. KA analyzed the data and revised the manuscript. SS and DO contributed to the conception and the design of the study. AB analyzed the data, wrote and revised the manuscript, and supervised the study. All authors contributed to the article and approved the submitted version.

## Funding

This work was supported by the Moroccan Ministry of Higher Education and Research and The National Center for Scientific and Technical Research (CNRST) through “PPR1” and Al-khawarizmi grants coordinated by AB.

## Conflict of Interest

The authors declare that the research was conducted in the absence of any commercial or financial relationships that could be construed as a potential conflict of interest.

## Publisher’s Note

All claims expressed in this article are solely those of the authors and do not necessarily represent those of their affiliated organizations, or those of the publisher, the editors and the reviewers. Any product that may be evaluated in this article, or claim that may be made by its manufacturer, is not guaranteed or endorsed by the publisher.

## Supplementary Material

Supplementary Figure 1 Clustering according to nearest neighbor. **(A, B)** distance between point based on IDH status and glioma subtype.

Supplementary Figure 2 Clustering according to Umap parameters. Correlation clustering based on similarity coefficients.

Supplementary Figure 3 Correlation matrix of 22 immune subsets and 7 immune checkpoints in Glioma patients. The numbers in the scale represent pearson correlation coefficient. Red cycle represents a negative correlation, blue cycle represents a positive correlation, and white boxes represent no correlation between two kinds of cells or checkpoint inhibitors.
