# Supplementary figures and images for: Prognostic Gene Expression Signature in Patients With Distinct Glioma Grades
Source: Front Immunol. 2021 Sep 1;12:685213. doi: 10.3389/fimmu.2021.685213 (PMC8448281; doi:10.3389/fimmu.2021.685213)

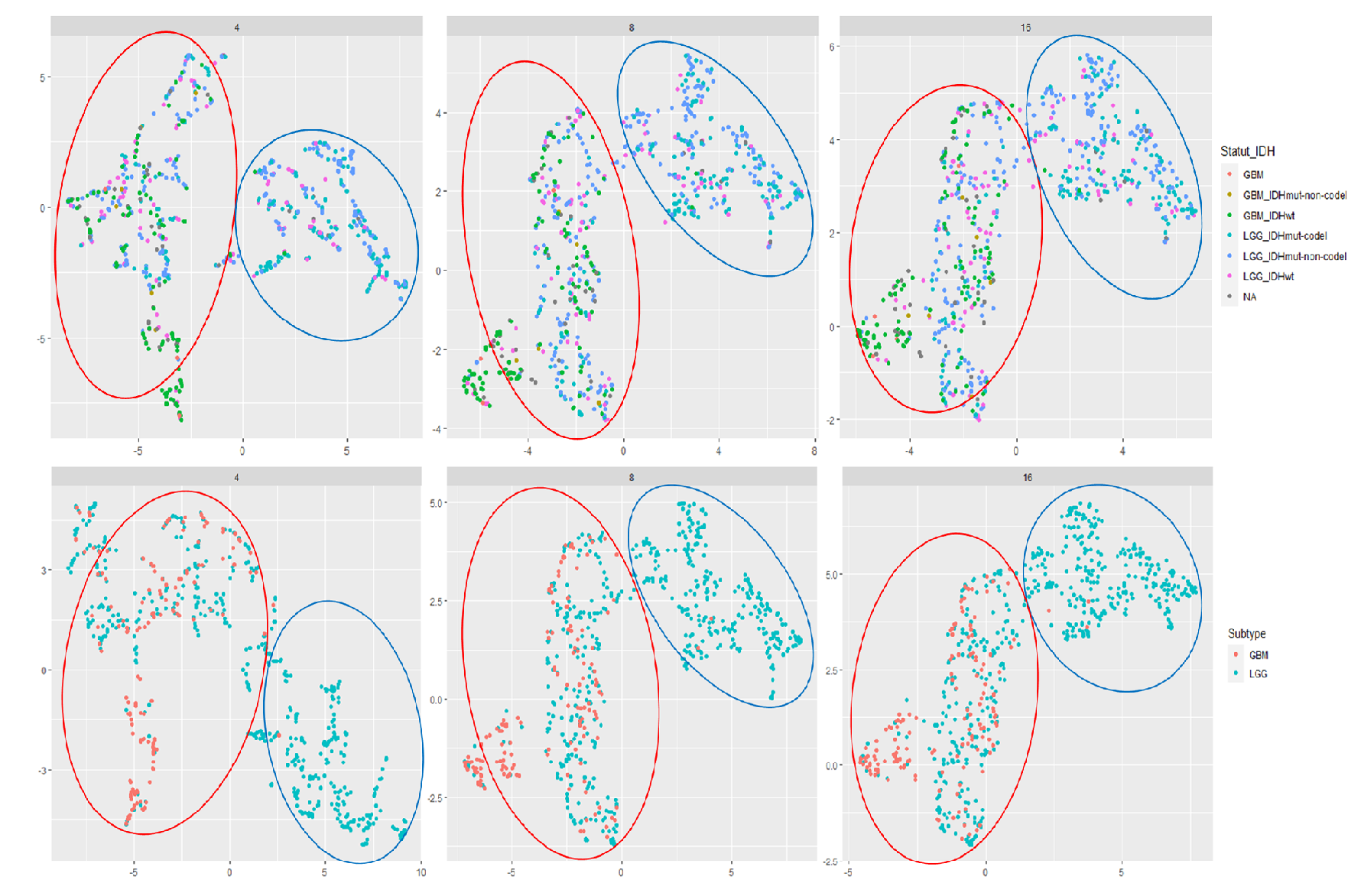

Supplement: Supplementary Figure 1 — Clustering according to nearest neighbor. (A, B) distance between point based on IDH status and glioma subtype. [file Image_1.tif]

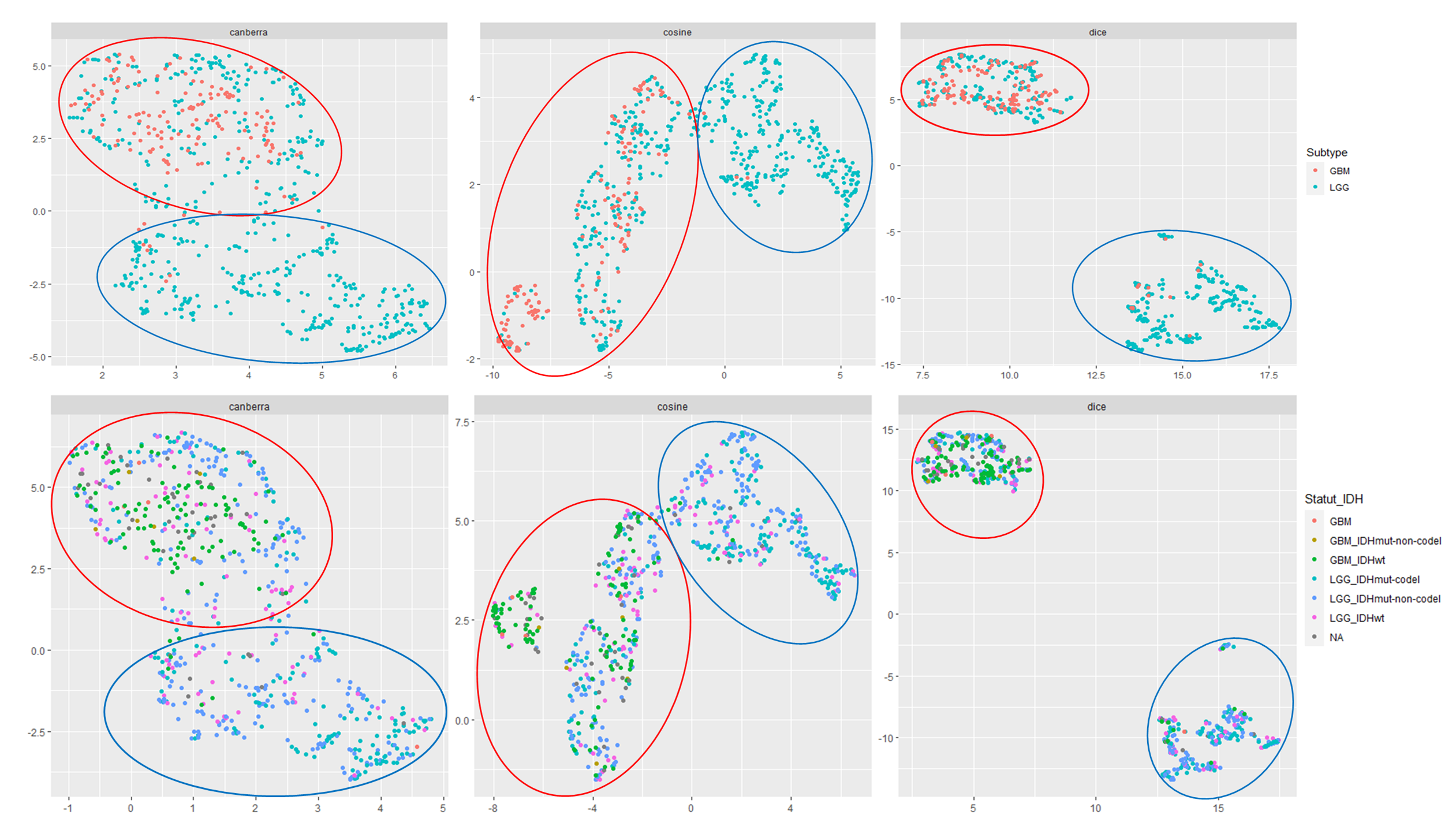

Supplement: Supplementary Figure 2 — Clustering according to Umap parameters. Correlation clustering based on similarity coefficients. [file Image_2.tif]

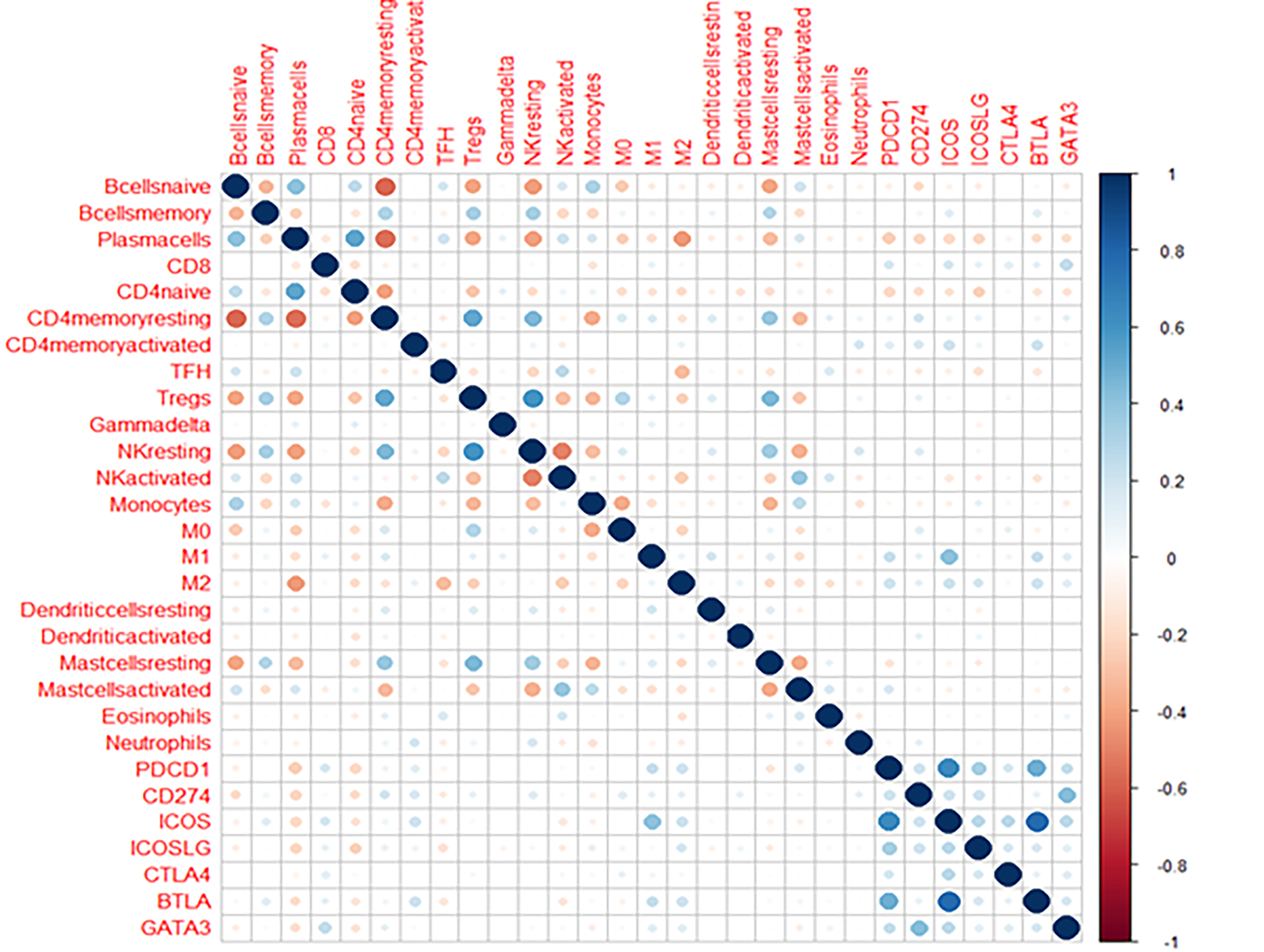

Supplement: Supplementary Figure 3 — Correlation matrix of 22 immune subsets and 7 immune checkpoints in Glioma patients. The numbers in the scale represent pearson correlation coefficient. Red cycle represents a negative correlation, blue cycle represents a positive correlation, and white boxes represent no correlation between two kinds of cells or checkpoint inhibitors. [file Image_3.tif]
